# Effects of insecticide use, host plant resistance, and nitrogen fertilization on the density of *Melanaphis sorghi* and the production of grain sorghum

**DOI:** 10.1038/s41598-025-96942-3

**Published:** 2025-04-09

**Authors:** Osariyekemwen Uyi, Xinzhi Ni, David Buntin, Michael D. Toews

**Affiliations:** 1 Department of Entomology, University of Georgia, 2360 Rainwater Rd., Tifton, GA 31793 USA; 2 Department of Animal and Environmental Biology, University of Benin, PMB 1154, Benin City, Nigeria; 3 Department of Zoology and Entomology, Faculty of Natural and Agricultural Sciences, University of the Free State, P.O. Box 339, Bloemfontein, 9300 South Africa; 4 Crop Genetics and Breeding Research Unit, USDA-ARS, 2747 Davis Road, Tifton, GA 31793 USA; 5 Department of Entomology, University of Georgia, 1109 Experiment St., Griffin, GA 30223 USA

**Keywords:** Nitrogen fertilization, Host plant resistance, insecticide efficacy, Aphid infestation, Foliar application, In-furrow application, Insect-plant interactions, Entomology, Agroecology

## Abstract

*Melanaphis sorghi* is a serious economically important pest of sorghum, *Sorghum bicolor* (L.), across the southern USA. Therefore, developing and refining integrated strategies that provide effective control is key to the management of this pest. The current study examined the influence of nitrogen (N) fertilization, sorghum cultivar and insecticide applications on *M. sorghi* and grain sorghum yield at Tifton, Georgia (31.5120° N, 83.6434° W). Field trials with three insecticide treatments (untreated control, flupyradifurone in-furrow at 117 g/ha, and flupyradifurone foliar at 73 g/ha), three nitrogen fertilization rates (25, 50 and 100 kg/ha) and two sorghum cultivars (resistant: DKS37-07 and susceptible: DKS53-53) were conducted on grain sorghum in the spring/summer of 2022 and 2023. Compared to the medium N fertilization, Low and high N fertilization supported higher aphid density and severity of infestation (cumulative insect days [CID]) on both the susceptible and resistant cultivars for both 2022 and 2023. Aphid density and severity of infestation on the susceptible sorghum cultivar (DKS53-53) were 3.4–4.8-fold greater than on the resistant cultivar (DKS37-07) for both low and high N fertilization plots in 2022. While a single foliar and in-furrow insecticide application significantly reduced infestations below the economic threshold across all treatment combinations in 2022, aphid populations were too low to warrant foliar application in 2023. Nitrogen fertilization was associated with improved yield as the high N fertilization preserved yield for both sorghum cultivars. Compared to untreated plots, in-furrow and foliar insecticide applications supported greater grain sorghum yield across all insecticide treatments only in 2022. The study suggests that manipulating N fertilization, utilizing resistant sorghum cultivars and in-furrow and foliar insecticide application can synergistically suppress aphid infestations and improve grain yield in sorghum production in southern USA.

## Introduction

Since the discovery of a new haplotype of the sorghum aphid, *Melanaphis sorghi* (Theobald) (Hemiptera: Aphididae) on grain sorghum, *Sorghum bicolor* (L.) (Poaceae), in Texas and Louisiana in the late summer of 2013^1–3^, this invasive multivoltine piercing-sucking insect has emerged as an important economic pest of sorghum in many southern states in the United States^4,5^. The invasion success of *M. sorghi* and its rapid spread to 25 states in the southern United States is due to the aphid’s high reproductive potential, dispersal capability and its potential to survive and reproduce in a wide range of climatic conditions, agroecosystems or uncultivated lands where the preferred or alternate host plants are abundant^4,6–9^. Although grain sorghum and forage sorghum are the preferred hosts of *M. sorghi*, this invasive sap-removing pest can survive on other types of non-crop sorghum during off season. *Melanaphis sorghi* is known to overwinter on Johnson grass, *Sorghum halepense* (L.), sudangrass (*Sorghum verticilliflorum*), Columbus grass (*Sorghum almum*; a hybrid between sorghum and Johnsongrass), and giant miscanthus, *Miscanthus sinensis* × *Miscanthus sacchariflorus* Greef & Deuter ex Hodkinson & Renvoize, in Alabama, Texas and Georgia^7,9,10^.

At high densities, sap feeding from the phloem tissues of the leaves, stems and panicles by nymphs and adults of *M. sorghi* causes leaf chlorosis, wilt, necrosis, loss of nutrient and sugars, stunted growth, prevention of panicle emergence or in extreme cases plant death in grain sorghum^4,6,11^. During feeding, *M. sorghi* produces copious amounts of honeydew which promotes the growth of sooty mold on leaf surface. The accumulation of sooty mold has been reported to impede photosynthesis of sorghum plants and may clog harvesting equipment^4,6^. At high densities, the damage caused by *M. sorghi* often translate into substantial yield loss (between 50 and 100%) in susceptible grain sorghum and forage sorghum cultivars^4,5,11–14^ leading to poor crop growth and development and significant economic losses for growers^4,15,16^.

To mitigate the damage caused by *M. sorghi* and improve sorghum yield, numerous research efforts have been focused on developing economic thresholds to guide foliar insecticide application, identifying resistant cultivars, evaluating planting sorghum date and other crop production practices in southern United States [e.g.^7,10,17–19^. While many studies that combined management strategies such as planting early in the season, application of foliar insecticides, planting resistant cultivars and manipulating natural enemy populations have been documented to mitigate aphid infestations and preserve grain sorghum yield^13,14,18–21^, further evaluation of factors and production practices that may influence *M. sorghi* infestations and the resulting yield losses are needed to refine integrated pest management (IPM) strategies in sorghum production systems.

Beyond manipulating planting date and timing insecticide application to limit the damage caused by *M. sorghi*, other agronomic practices such as manipulating nitrogen fertilization may be an important driver of the population dynamics of this invasive pest. Nitrogen fertilization is known to influence plant growth and quality; an increase in nitrogen fertilization generally accelerates growth and improves plant quality^22–24^. Although studies have demonstrated, through their contrasting results, that the relationships between nitrogen levels in host plants and phytophagous insect performance are not simple^25–28^, the general rule is clearly a positive association^24,29,30^—be it with modifications. Increased nitrogen in plants has often been linked with increased phytophagous insect survival, fecundity, and population growth rates^24,29–31^ in insects including aphids^28,32–34^. Understanding how nitrogen fertilization independently or cumulatively interacts with other factors to influence the population dynamics of *M. sorghi* could help refine IPM strategies in sorghum production. Field studies on the influence of nitrogen fertilization on *M. sorghi* infestations and the resulting sorghum yield are still scarce (but see^35^). Although Wilson et al.^35^ investigated the effect of nitrogen fertilization and foliar insecticide application on *M. sorghi* infestations and resulting sorghum yield, the authors did not consider in-furrow insecticidal application in their study. The recent data on the efficacy of in-furrow applications of flupyradifurone in concert with other agronomic practices in managing *M. sorghi*^19^ in grain sorghum has greatly advanced the development of IPM efforts against this pest. Therefore, research that integrates in-furrow insecticide application and nitrogen fertilization as well as other agronomic practices may further improve IPM strategies against *M. sorghi*. Knowledge of manipulating fertilizer and insecticide applications among commercial varieties in the control of *M. sorghi* can enable growers to make informed decisions on how to effectively manage this pest. The objectives of this research were to determine the effects of nitrogen fertilization, grain sorghum cultivar, and insecticide applications on *M. sorghi* infestations and sorghum yield in Tifton, southern Georgia.

## Materials and methods

### Experimental design, agronomic practices, and nitrogen fertilization levels

The impact of three nitrogen fertilizations (25, 50 and 100 kg/ha), two grain sorghum cultivars (resistant: DKS37-07, Dekalb, Bayer CropScience, St. Louis, MO; susceptible: DKS53-53, Dekalb, Bayer CropScience, St. Louis, MO), and two different methods of insecticide application (in-furrow and foliar applications) on *M. sorghi* densities and grain sorghum yield were evaluated over two growing seasons (2022 and 2023) at Tift County GA (31.5120° N, 83.6434° W). DKS37-07 is a medium early sorghum aphid tolerant hybrid with bronze grain, excellent yield potential, strong seedling vigor, high test weight, very good stalk strength across a wide geography. In spring of 2022 (May 24th) and 2023 (May 19th), plots of grain sorghum (DKS37-07 and DKS53-53) with different nitrogen fertilization treatments were established. A 3 × 2 × 3 factorial experiment arranged in a split-split plot design with three levels of nitrogen fertilization (25, 50 and 100 kg/ha) as the main plot factor, two sorghum cultivars (DKS53-53 vs DKS37-07) as the subplot factor, and three insecticide application levels (untreated, in-furrow, and foliar insecticide applications) as the sub-subplot factor, in three replications was used. A total of 54 plots were used for the experiment. Soil testing showed that phosphorus, potassium, calcium, magnesium, manganese and zinc levels as well as soil pH were even and similar across the plots before the addition of nitrogen fertilization as a treatment in our study (supplementary Table 1). Before planting, 25 kg/ha of nitrogen fertilization was applied and incorporated into all plots. After planting, plots received an additional 25 kg/ha of nitrogen on the medium nitrogen fertilization treatment, and 75 kg/ha of nitrogen on the high nitrogen fertilization treatment whereas the low nitrogen fertilization treatment did not receive any additional nitrogen fertilizer. Nitrogen was applied 3–4 weeks after planting in both 2022 and 2023. All plots measured 4 rows by 12.2 m (0.91-m row spacing) and were planted using a vacuum planter (Monosem® Inc., Edwardsville, KS) at a density of 186,186 plants per ha. All production practices were consistent with recommendations for commercial production of sorghum in southern USA^36^. Pictures of grain sorghum plots showing plant response to the different N. fertilization treatments are shown in supplementary Fig. 1.

### Insecticide treatment and aphid infestation assessment

The three insecticide treatments were untreated, 117 g/ha flupyradifurone in-furrow, and 73 g/ha flupyradifurone + adjuvant as a foliar flupyradifurone (Sivanto Prime, Bayer CropScience, Rhein, Germany) application. The in-furrow insecticide was applied at planting in selected plots while the foliar application was applied when aphid population reached the economic threshold of 50 aphids per lower leaf (for rationale, see^13^). For the in-furrow insecticide application, a microjet applicator applied a total of 57.5 L of diluted insecticide per ha in the open furrow in front of the disk closer and press wheel. Designated foliar plots received a one-time application of flupyradifurone at 73 g/ha administered using a self-propelled sprayer equipped with hollow cone nozzles (model TXVS-8, TeeJet Technologies, Spraying Systems Co., Glendale Heights, IL). Foliar applications were delivered in a spray volume of 93.5 L/ha. In 2023, aphid populations never exceeded the treatment threshold across grain sorghum cultivars, hence no foliar insecticide application was made. *Melanaphis sorghi* infestations were estimated by enumerating aphids, regardless of age, on each sampled leaf. Assessments started four weeks after planting and continued until the grain reached the hard dough stage; sugarcane aphids were counted on 6 lower and 6 upper leaves of 12 randomly selected sorghum plants per plot each week. To simultaneously account for aphid abundance and duration of infestations, aphid counts from top and bottom leaves were converted to cumulative insect days (CID) on a per plot basis following the methods of Ruppel^37^. Briefly, aphid days were calculated for each sampling interval as the mean density of two consecutive sample dates multiplied by the length of the interval between the dates in days. These values accumulated over the entire sampling period in each year, providing a cumulative estimate of aphid infestation intensity for each plot.

### Harvest

When the grain dried in the field to a moisture content of 15% or less, the center two rows from each plot were harvested using a self-propelled combine. The middle 2-rows of each plot were harvested for yield on September 27th in 2022 and August 31st in 2023. For comparison purposes, all plots were adjusted to a common 14% moisture content and extrapolated to kg of grain per ha.

### Total rainfall and temperature data

Total rainfall data from the first day of aphid detection until aphid population crash in grain sorghum over two growing seasons (2022 and 2023) was obtained from an automated weather station located 2.8 km from the plots (www.georgiaweather.net). Minimum air temperature data was also obtained from the weather station covering the time period January 2021 through March 2023; this dataset was used to ascertain whether prior wintery temperatures affected aphid migration from overwintering hosts to sorghum, and subsequently its population densities on sorghum crop in the spring/summer.

### Data analysis

Because no foliar insecticide treatments were administered in 2023, both CID and grain yield data from the foliar plots were combined with data from the untreated plots. Therefore, only two insecticide treatment methods (in-furrow insecticide treatment and untreated) were considered for analysis in 2023. The effect of nitrogen fertilization, grain sorghum cultivar and insecticide applications on CID was log transformed and analyzed using a Generalized Linear Model (GLM) (assuming normal distribution with an identity link function). Our models included CID as dependent variable with nitrogen fertilization, grain sorghum cultivar and insecticide application methods and all interaction (N fertilization × cultivar × insecticide treatment) as fixed effects and plot as a random effect. When the overall results were significant in the GLM analysis, differences among means were compared using the sequential Bonferroni test. The effect of nitrogen fertilization, grain sorghum cultivar, and insecticide applications on grain sorghum yield was evaluated using a Generalized Linear Model (GLM) (assuming Gaussian distribution with an identity link function). When the overall results were significant, the differences in means were compared using the Tukey’s Honest Significant Difference (HSD) test. All analyses were performed using IBM SPSS Statistical software version 20.0 (SPSS, Chicago, IL, USA).

## Results

### Aphid densities

**Fig. 1:**
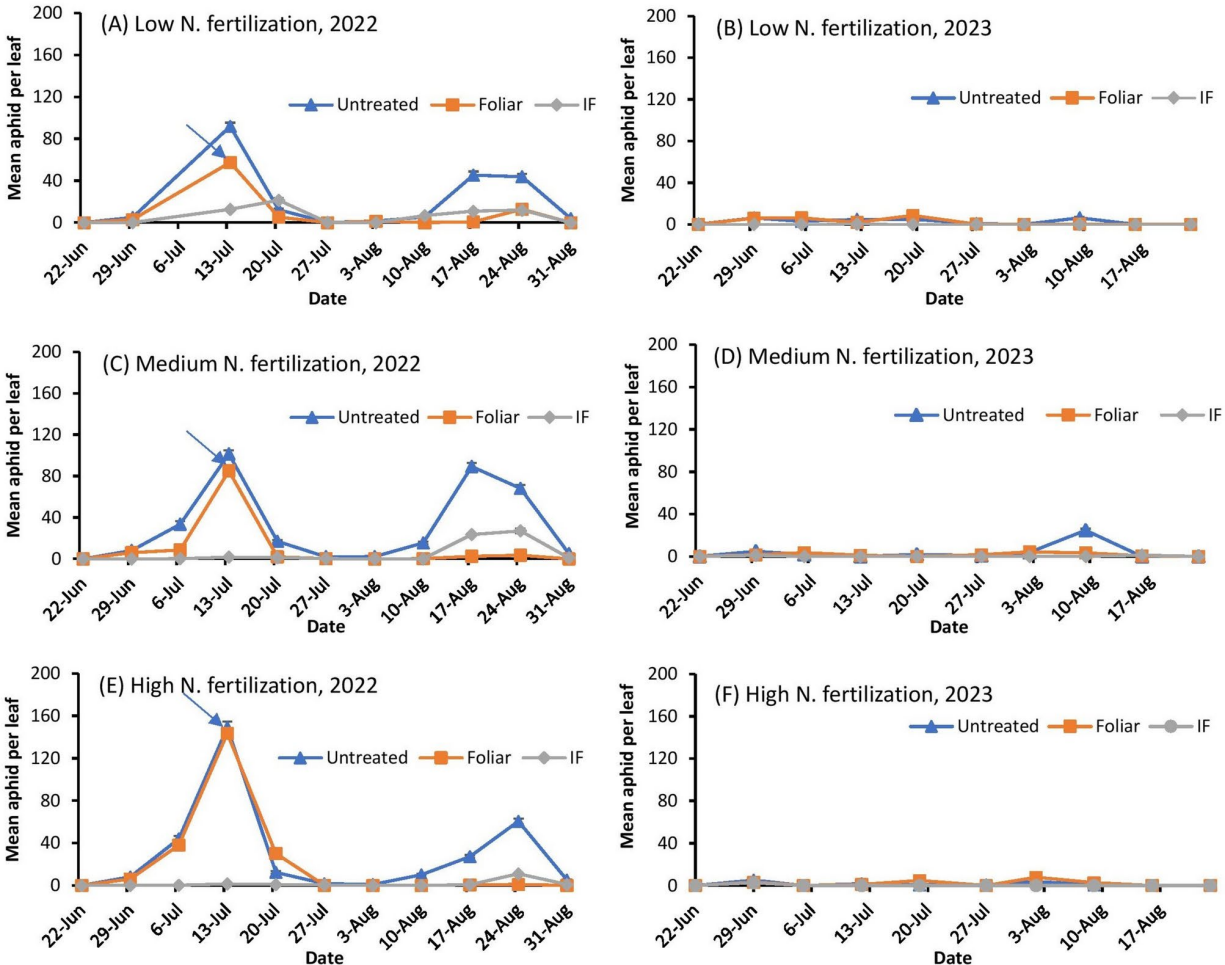
Mean number of *Melanaphis sorghi* on bottom leaves for low (**A** and **B**), medium (**C** and **D**) and high (**E** and **F**) N fertilization levels in **resistant grain sorghum (DKS37-07)** treated with flupyradifurone in-furrow (IF) or foliar application in 2022 and 2023. The arrow indicates the timing of foliar insecticide application. The economic threshold of 50 aphids per lower leaf was used for this study.

**Fig. 2:**
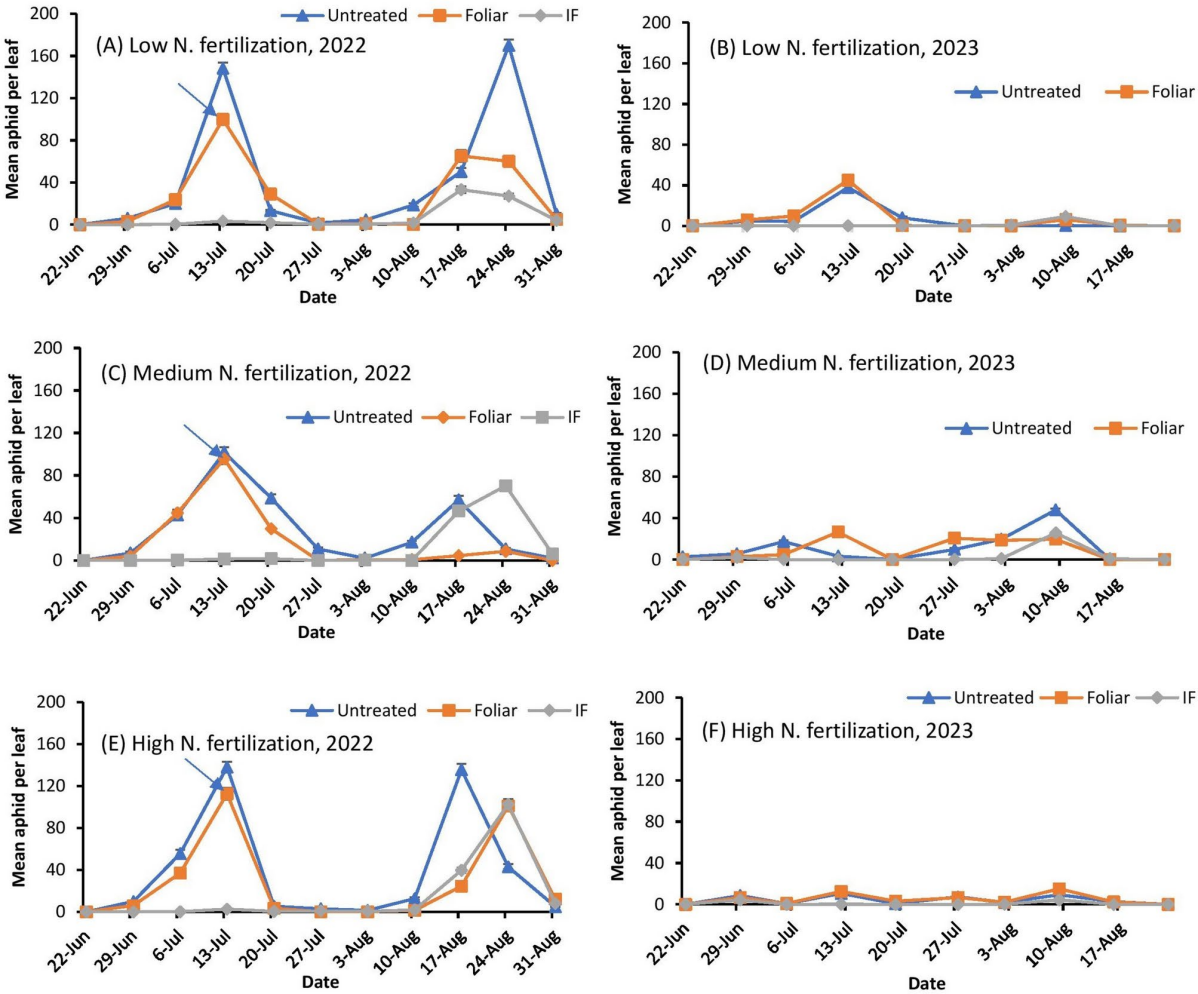
Mean number of *Melanaphis sorghi* on bottom leaves for low (**A** and **B**), medium (**C** and **D**) and high (**E** and **F**) N fertilization levels in **susceptible grain sorghum (DKS53-53)** treated with flupyradifurone in-furrow (IF) or foliar application in 2022 and 2023. The arrow indicates the timing of foliar insecticide application. The economic threshold of 50 aphids per lower leaf was used for this study.

In 2022, aphids were first found on June 22nd (29 days after planting) across all nitrogen fertilization treatments on both the susceptible (DKS53-53) and the resistant (DKS37-07) grain sorghum cultivars (Figs. 1A, C, E and 2A, C, E). Aphid populations were higher on the susceptible cultivar (DKS53-53) compared to plots with the resistant (DKS37-07) cultivar. Similarly, the high and low nitrogen fertilization treatment supported higher aphid numbers when compared to the medium nitrogen treatment. Aphid populations on both grain sorghum cultivars exceeded the treatment threshold (50 aphids per lower leaf) in the untreated plots within three weeks of first detection. For both cultivars, foliar application to selected plots occurred on July 12th and immediately brought the infestation below threshold (Figs. 1A, C, E and 2A, C, E). For the resistant cultivar, aphid numbers in the untreated plots and foliar application plots declined concurrently across all nitrogen fertilization treatments but a population resurgence of this pest was only experienced in the untreated plots (Fig. 1A, C, E). Following a population decline to near zero in all three nitrogen fertilization treatments in plots with the susceptible cultivar, aphid populations steadily increased from August 3^rd^ and exceeded the treatment threshold in all treatment plots (including in-furrow) except in the medium nitrogen fertilization plots where aphid population constantly remained at near zero until the grains were harvested (Figs. 2A, C, E). In-furrow insecticide application suppressed aphid population to a near zero level in the resistant cultivar across all nitrogen fertilization treatments.

Aphid densities in 2023 were generally very low when compared to 2022. Aphids first appeared on June 22nd (24 d after planting) across all nitrogen fertilization treatments in both the susceptible (DKS53-53) and the resistant (DKS37-07) grain sorghum cultivar cultivars (Figs. 1B, D, F and 2B, D, F). Aphid populations never exceeded the treatment threshold in the treatment plots on both grain sorghum cultivars to warrant foliar insecticide application.

### Cumulative insect days

**Fig. 3:**
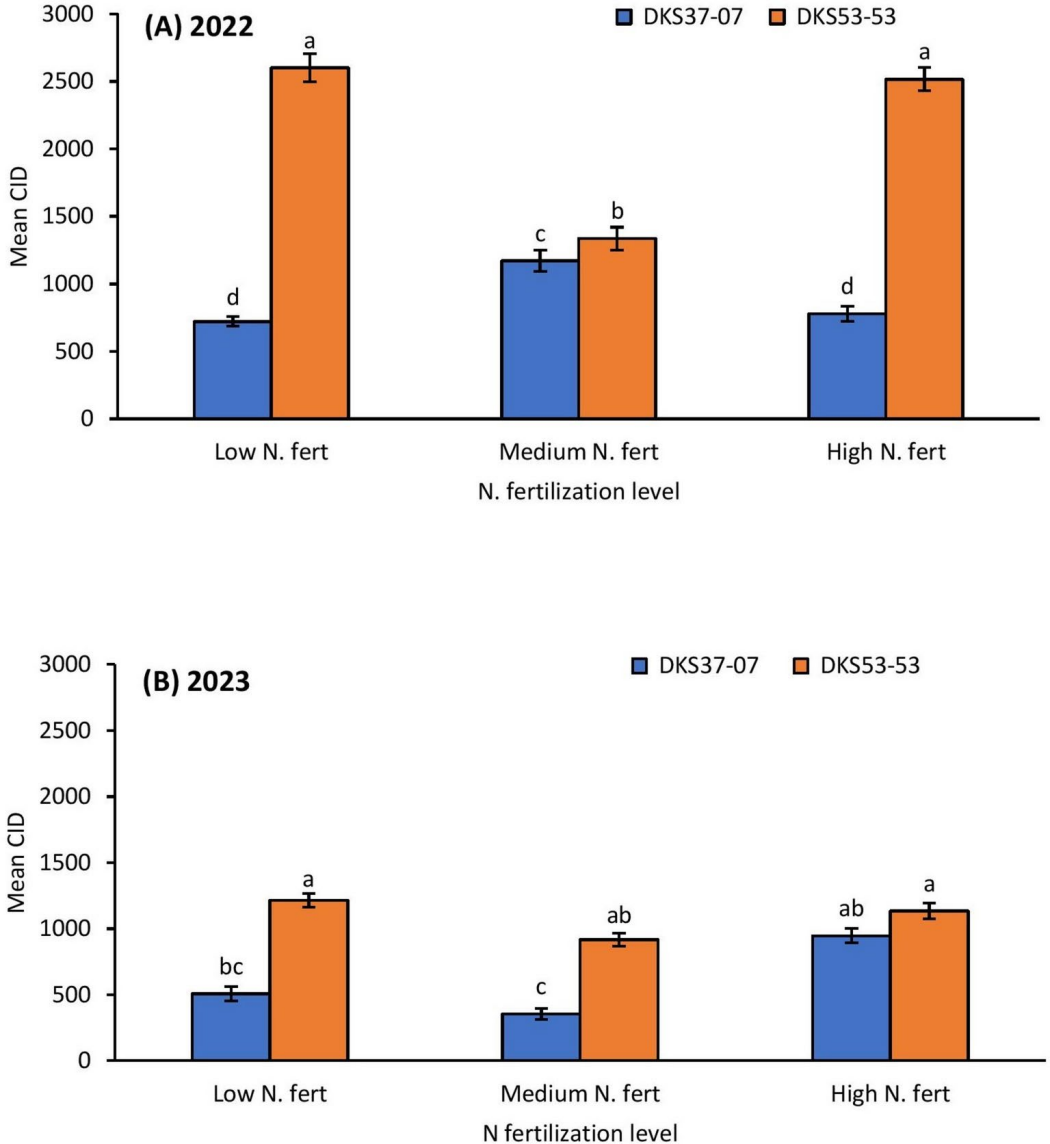
Mean (± SE) cumulative insect days (CID) as affected by N fertilization × sorghum cultivar in 2022 (**A**); and 2023 (**B**). Means with different letters are significantly different (sequential Bonferroni test, *P* < 0.05) among all three N fertilization levels and two sorghum cultivars.

**Fig. 4:**
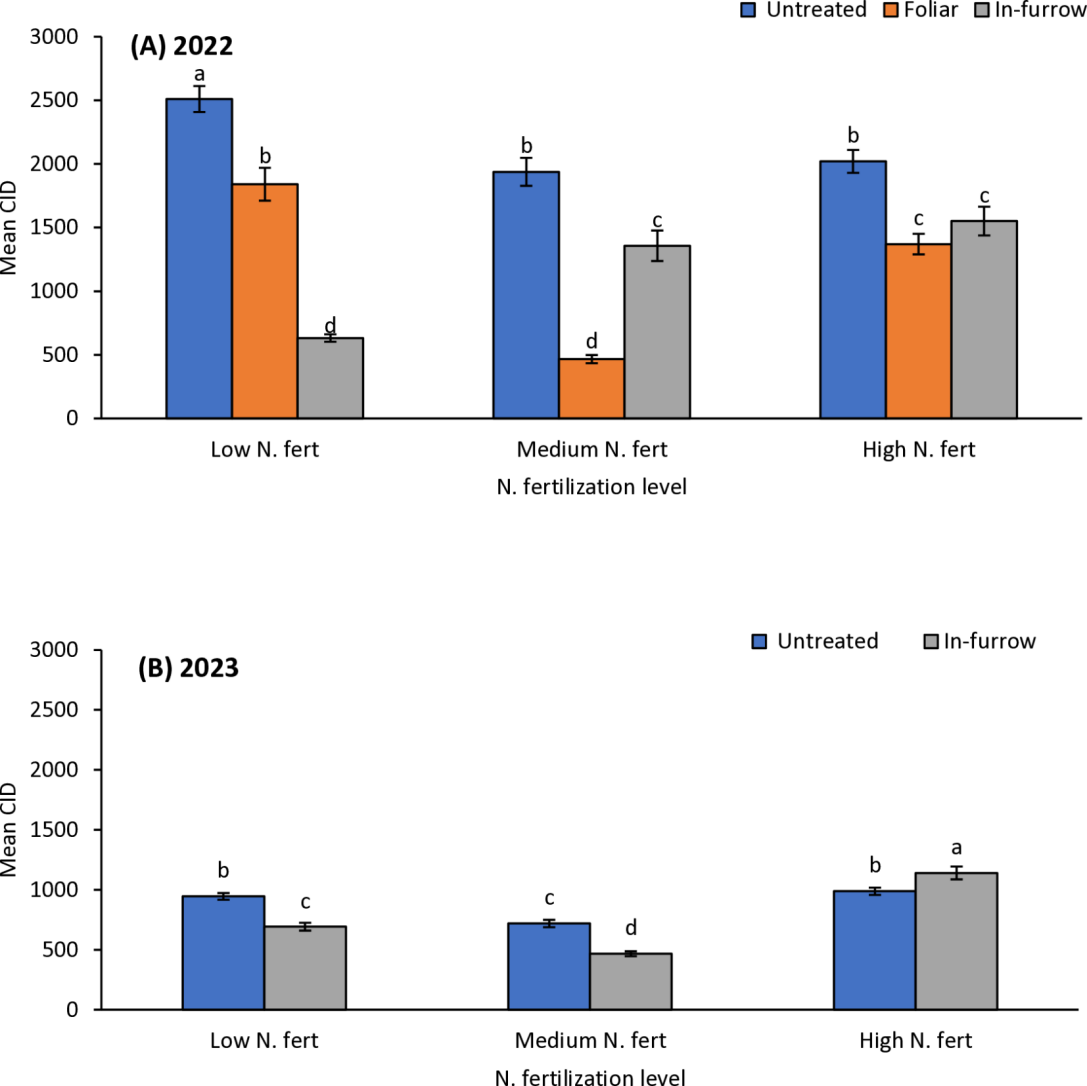
Mean (± SE) cumulative insect days (CID) as affected by N fertilization × insecticide treatment in 2022 (**A**); and 2023 without foliar insecticide application (**B**). Means with different letters are significantly different (sequential Bonferroni test, *P* < 0.05) among all three N fertilization levels and insecticide treatments.

**Fig. 5:**
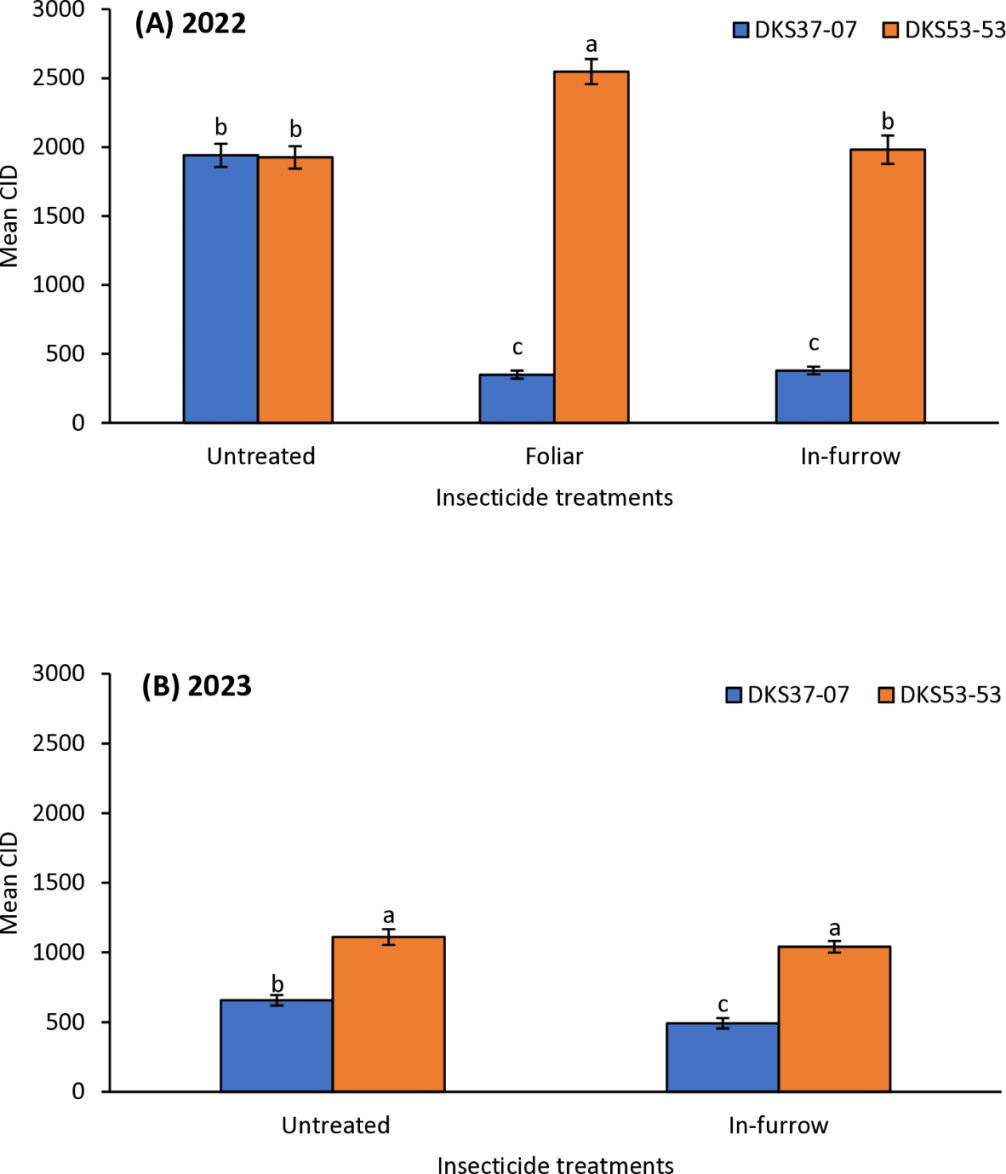
Mean (± SE) cumulative insect days (CID) as affected by insecticide treatment × sorghum cultivar in 2022 (**A**); and 2023 without foliar insecticide application (**B**). Means with different letters are significantly different (sequential Bonferroni test, *P* < 0.05) among all three insecticide treatments and two sorghum cultivars.

*Melanaphis sorghi* infestations as indicated by CID were significantly influenced by nitrogen fertilization, grain sorghum cultivar and insecticide application in 2022 (nitrogen fertilization: χ^2^ = 42.594, *P* = 0.0001; sorghum cultivar: χ^2^ = 474.692, *P* = 0.0001; insecticide application: χ^2^ = 115.741, *P* = 0.0001) and 2023 (nitrogen fertilization: χ^2^ = 51.399, *P* = 0.0001; sorghum cultivar: χ^2^ = 87.002, *P* = 0.0001; insecticide application: χ^2^ = 4.753, *P* = 0.0290) (Table 1; Figs. 3, 4 and 5). Generally, grain sorghum plots with low and high nitrogen fertilization supported higher *M. sorghi* infestations compared to the medium nitrogen fertilization treatments in 2022 and 2023 (Table 1; Fig. 3). Aphid infestation was higher on the susceptible grain sorghum cultivar (DKS53-53) across all the three nitrogen fertilization treatments in both 2022 and 2023 (Fig. 3A and B). The severity of infestation on the susceptible grain sorghum cultivar (DKS53-53) was 2.5- to 3.8-fold greater than the values on the resistant cultivar (DKS37-07). The CID values in the susceptible cultivar did not differ between the low and high nitrogen fertilization treatments in 2022 and 2023 (Fig. 3A and B). Aphid infestations (CID) were highest in untreated plots across all three nitrogen fertilization treatments in 2022 and 2023 compared to treated plots except in the high nitrogen fertilization treatment in 2023 where CID values was higher in the in-furrow treatment than the untreated CID values (Fig. 4A and B). Among the in-furrow treatments, the medium and high nitrogen fertilization plots supported more aphids compared to low nitrogen fertilization plots in 2022 (Fig. 4A); whereas in 2023, high and low nitrogen fertilization treatments supported higher CID values compared to the medium nitrogen fertilization treatments (Fig. 4B). In 2022, CID values were between 40 to 50% higher in untreated plots relative to insecticide treated plots across all three nitrogen fertilization treatments (Fig. 4A). However, in 2023, CID values were only 27% and 35% higher in untreated plots relative to in-furrow insecticide treated plots in the low and medium nitrogen fertilization treatments, respectively (Fig. 4B). In both 2022 and 2023, in-furrow insecticide applications consistently resulted in the lowest aphid infestation levels, except the high N treatment plots in 2023 (Fig. 4B). It is worth noting that aphid population was very low though. In both insecticide treatments, the susceptible cultivar relative to the resistant cultivar had higher severity of aphid infestation in 2022, however, CID values in the untreated plots did not differ between the two grain sorghum cultivars (Fig. 5A). In 2023, aphid infestation on the susceptible cultivar relative to the resistant cultivar was higher in both treatment and untreated plots (Fig. 5B).

**Table 1:** Generalized linear model (GLM) results for effects of nitrogen fertilization, grain sorghum cultivar, insecticide application and all interactions on cumulative insect days (CID).

Effect	2022			2023		
	d.f	Wald χ^2^	*P*	d.f	Wald χ^2^	*P*
Intercept	1	2760.112	0.0001	1	942.874	0.0001
Nitrogen	2	42.954	0.0001	1	51.399	0.0001
Cultivar	1	474.692	0.0001	1	87.002	0.0001
Insecticide	2	115.741	0.0001	1	4.753	0.0290
N × cultivar	2	180.040	0.0001	2	7.712	0.0210
N × insecticide	4	300.222	0.0001	2	12.605	0.0020
Cultivar × insecticide	2	260.589	0.0001	1	0.779	0.3780
N × cultivar × insecticide	4	250.444	0.0001	2	15.728	0.0001

### Grain sorghum yield

**Fig. 6:**
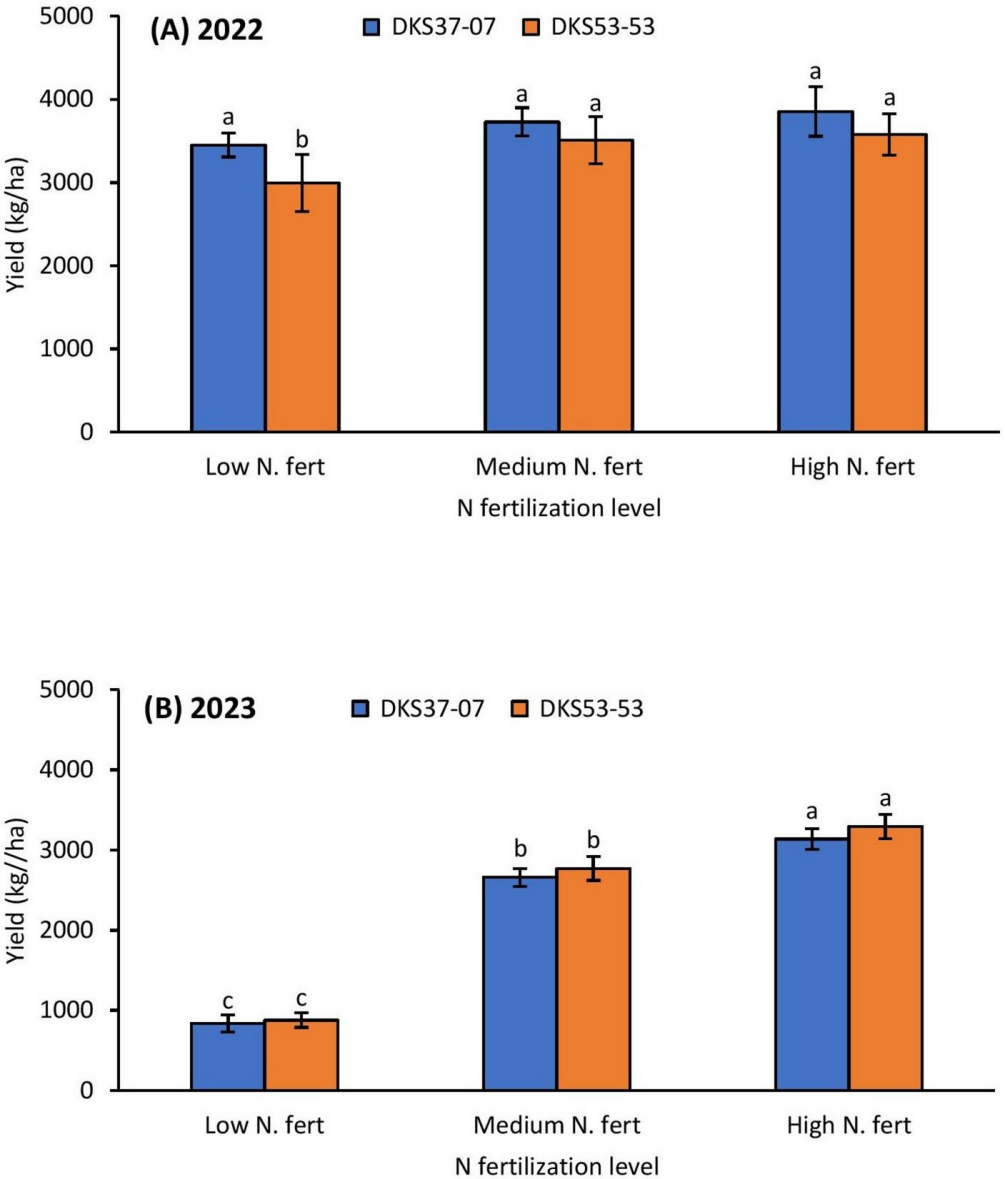
Mean (± SE) grain sorghum yield (kg/ha) as affected by N fertilization × sorghum cultivar in 2022 (**A**); and 2023 (**B**). Means with different letters are significantly different (sequential Bonferroni test, *P* < 0.05) among all three N fertilization levels and two sorghum cultivars.

**Fig. 7:**
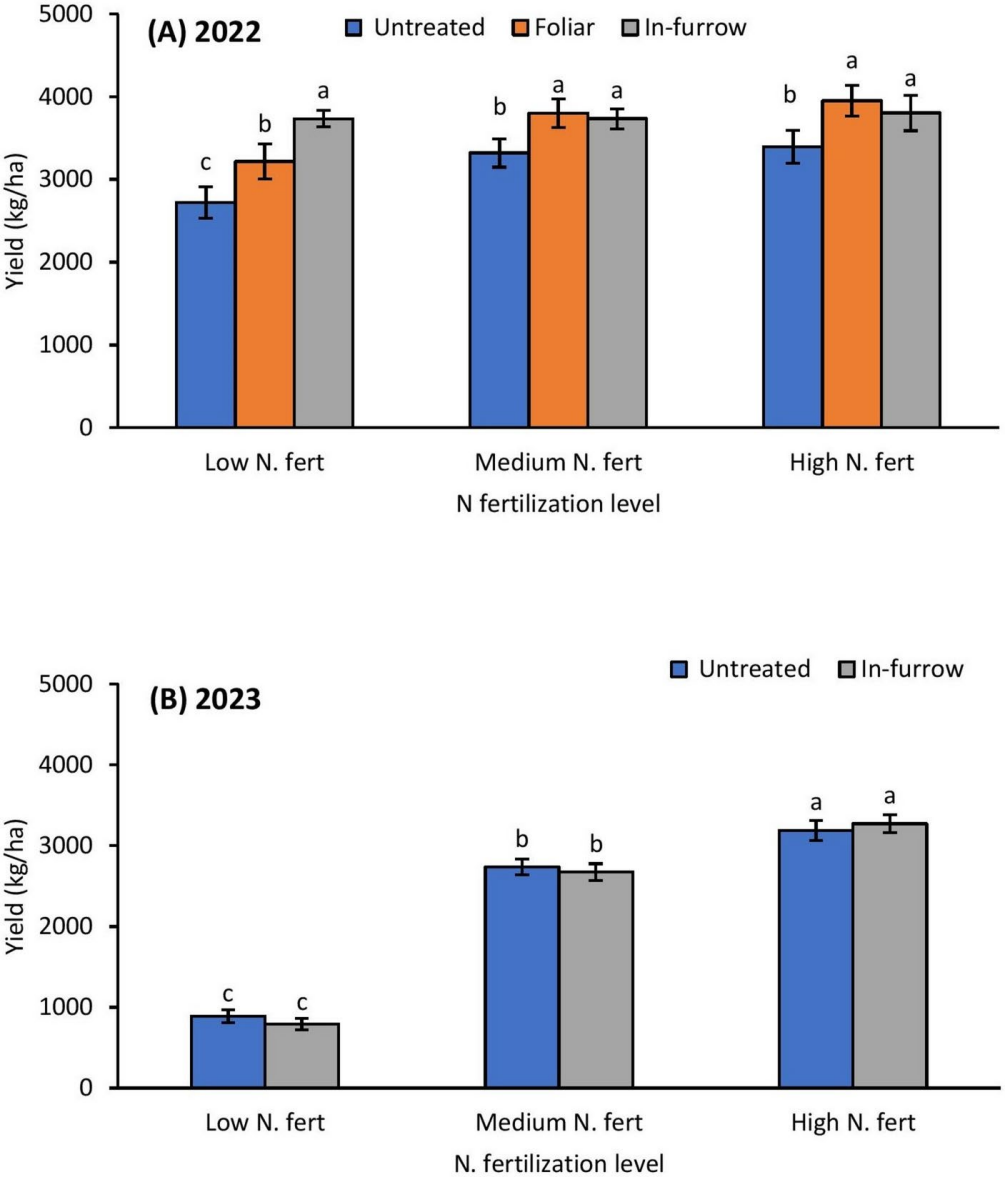
Mean (± SE) grain sorghum yield (kg/ha) as affected by N fertilization × insecticide treatments in 2022 (**A**); and 2023 without foliar insecticide application (**B**). Means with different letters are significantly different (sequential Bonferroni test, *P* < 0.05) among all three N fertilization levels and insecticide treatments.

In 2022, grain yield varied with nitrogen fertilization (χ^2^ = 6.515, *P* = 0.0380), grain sorghum cultivar (χ^2^ = 3.805, *P* = 0.0540) and insecticide application (χ^2^ = 10.922, *P* = 0.0040). There was no significant interaction between the various treatment combinations (Table 2). In 2023, grain sorghum yield was significantly influenced by nitrogen fertilization (χ^2^ = 423.143, *P* = 0.0001) but did not differ between the cultivars (χ^2^ = 1.641, *P* = 0.2000) or insecticide treatment (χ^2^ = 0.068, *P* = 0.7940). As with the case in 2022, no significant interactions between treatment combinations were observed (Table 2). Generally, high and medium nitrogen fertilization preserved yield by 10% compared to the low nitrogen fertilization treatment in 2022 (Fig. 6A). Although sorghum yield did not differ between the susceptible and resistant cultivars in both the medium and the high nitrogen fertilization treatments, the resistant cultivar produced better yield in the low nitrogen fertilization treatments in 2022 (Fig. 6A). In 2023, high and medium nitrogen fertilization supported better yield compared to the low nitrogen fertilization treatment (Fig. 6B). Grain sorghum yield did not vary with sorghum cultivar across all nitrogen fertilization treatments in 2023 with low aphid infestation (Fig. 6B). In 2022, the insecticide treated plots (relative to the untreated plots) supported improved grain yield across all the three nitrogen fertilization treatments, but the effect was higher in the medium and high nitrogen fertilization treatments (Fig. 7A). In 2023, a similar trend was observed under low aphid infestation, high and medium nitrogen fertilization supported better yield compared to the low nitrogen fertilization treatment (Fig. 7B). Grain sorghum yield was not influenced by insecticide application across all nitrogen fertilization treatments in 2023 (Fig. 7B).

**Table 2:** Generalized linear model (GLM) results for effects of nitrogen fertilization, grain sorghum cultivar, insecticide application and all interactions on grain yield (kg/ha).

Effect	2022			2023		
	d.f	Wald χ^2^	*P*	d.f	Wald χ^2^	*P*
Intercept	1	1784.936	0.0001	1	2053.398	0.0001
Nitrogen	2	6.515	0.0380	2	423.143	0.0001
Cultivar	1	3.805	0.0540	1	1.641	0.2000
Insecticide	2	10.922	0.0040	1	0.068	0.7940
N × cultivar	2	0.358	0.8360	2	0.218	0.8970
N × insecticide	4	2.577	0.6310	2	0.639	0.7270
Cultivar × insecticide	2	0.890	0.6410	1	0.656	0.3780
N × cultivar × insecticide	4	2.999	0.5580	2	0.359	0.8360

**Fig. 8:**
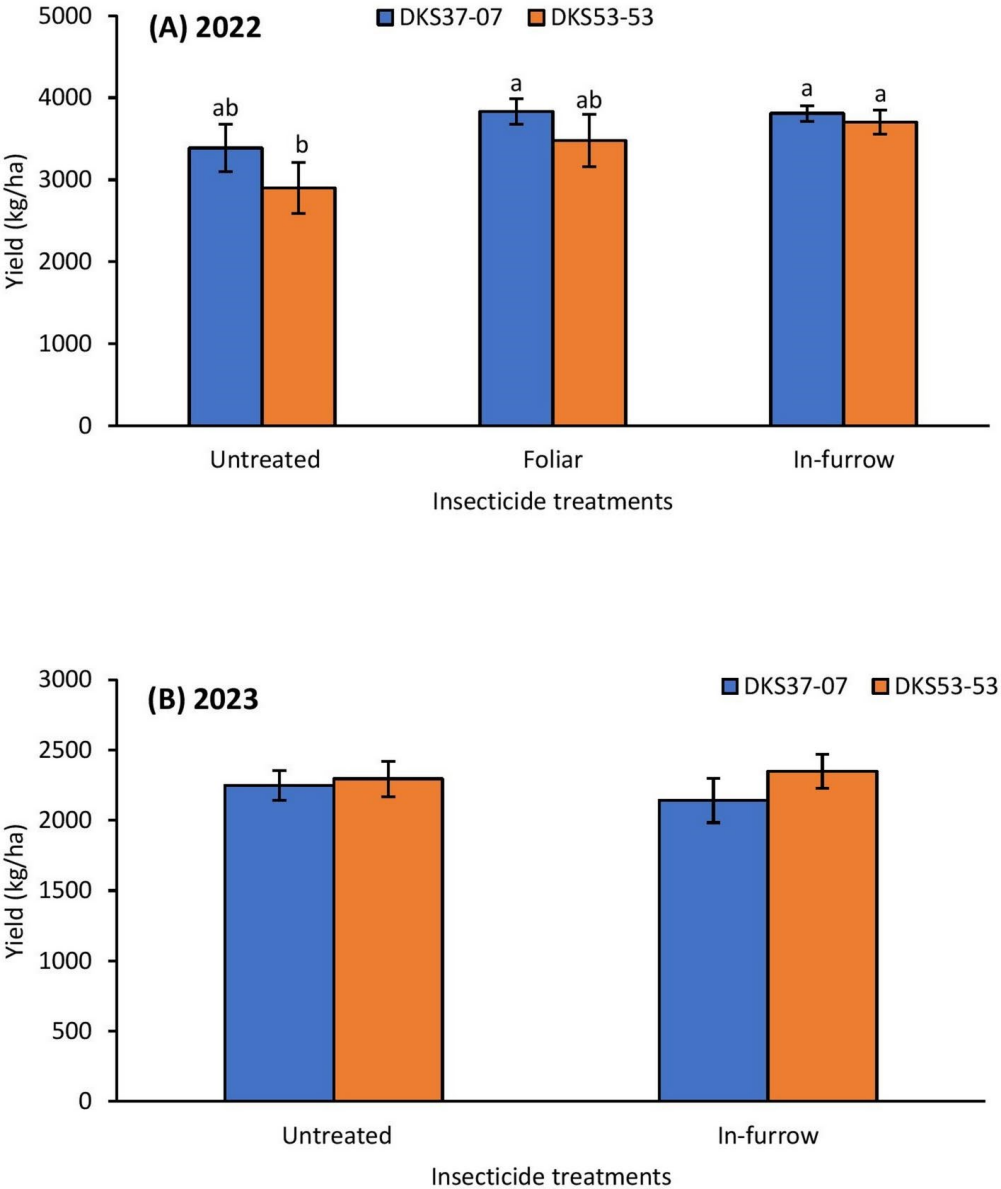
Mean (± SE) grain sorghum yield (kg/ha) as affected by insecticide treatment × sorghum cultivar in 2022 (**A**); and 2023 without foliar insecticide application (**B**). Means with different letters are significantly different (sequential Bonferroni test, *P* < 0.05) among all three insecticide treatments and two sorghum cultivars.

In addition, the grain yield was also compared between the two cultivars. In 2022, the insecticide treated resistant sorghum cultivar produced numerically higher yield when compared to the untreated (Fig. 8A), although the data were not statistically significant. Sorghum yield in the untreated and foliar insecticide treated plots was slightly higher on the resistant cultivar compared to the susceptible cultivar (Fig. 8A) in 2022. In 2023, under low aphid infestation, insecticide application and grain sorghum cultivar did not appear to have any significant effect on grain sorghum yield (Fig. 8B).

### Weather (Total rainfall and temperature) events and sizes range

**Fig. 9:**
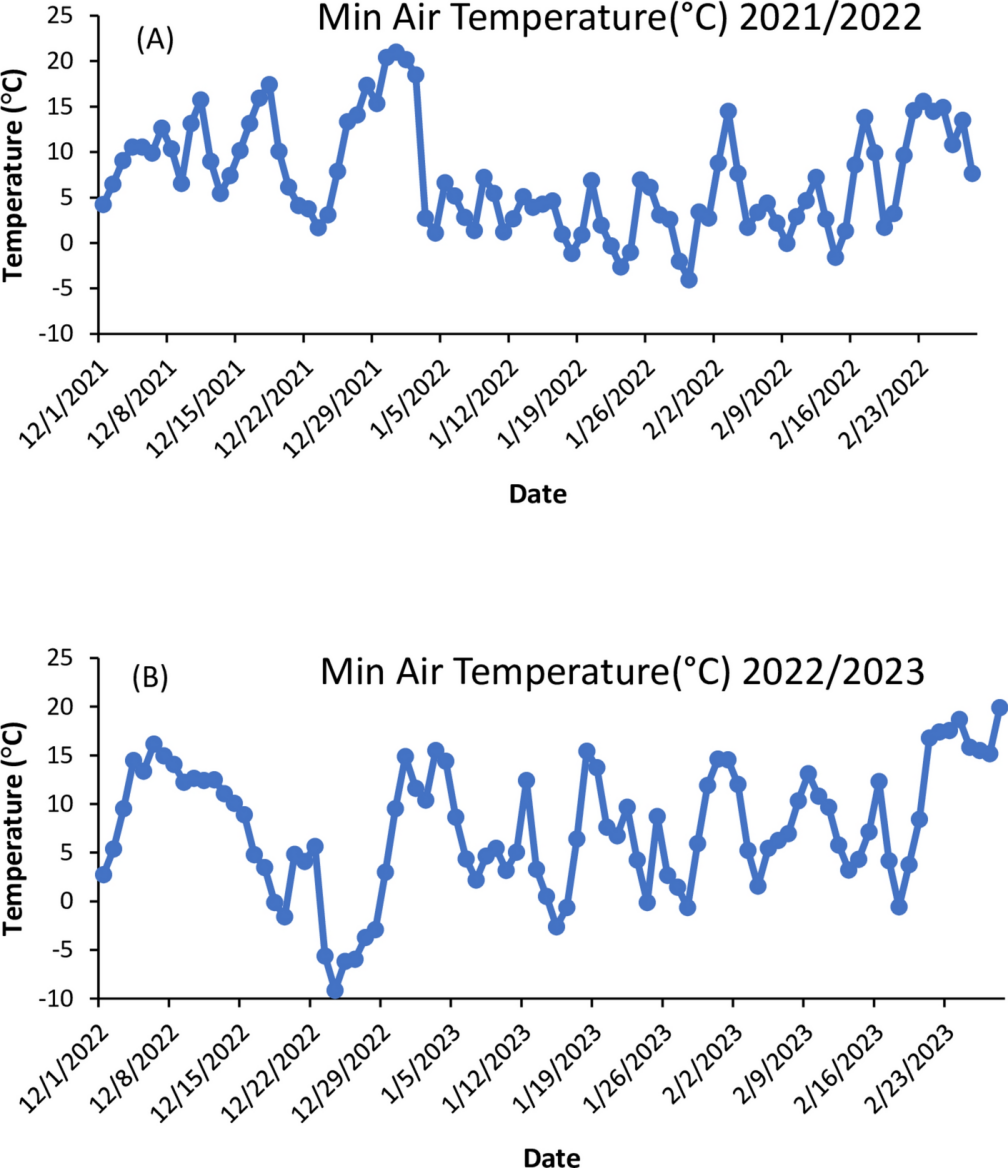
Minimum air temperature (°C) from December 2021 to March 2022 (**A**); and from December 2022 to March 2023 (**B**).

Cumulative rainfall from the first day of aphid detection until aphid population crash was 391 mm in 2022 and 251 mm in 2023 (Table 3). There were 12 rainfall events of ≥ 11 mm in 2022 that amounted to 286 mm, whereas in 2023 there were nine rainfall events of ≥ 11 mm that amounted to 197 mm. Minimum air temperature did not fall below freezing in the winter month of December 2021 (Fig. 9A). Between late January and mid-February 2022, minimum temperature dropped below zero (− 1 to − 4 °C) but did not persist for more than two days (Fig. 9A). Minimum air temperature dropped to − 6 °C on 23rd December 2022 and further dropped to − 9 °C and remained below zero for 6 days before increasing to 3 °C on 29th December 2022 (Fig. 9B).

**Table 3:** Rainfall events from June to September of 2022 and 2023 at Tifton, GA. The period encompasses the first day of aphid detection until grain sorghum was harvested.

Amount (mm)	2022		2023	
	Frequency	Cumulative amount (mm)	Frequency	Cumulative amount (mm)
≤ 2	13	10.92	13	9.91
3–5	3	10.41	4	15.49
6–10	11	82.80	4	28.96
11–15	5	66.80	3	37.84
16–20	1	17.78	1	17.78
21–30	4	100.58	4	101.35
31–40	1	36.32	1	39.88
≥ 40	1	65.28	–	–
Total	39	390.88	30	251.22

## Discussion

Given the economic significance of this invasive pest, further evaluation of factors and production practices that provide effective control that limits *M. sorghi* infestations and improve resulting grain sorghum yield are needed to refine integrated pest management (IPM) strategies in sorghum production systems^5,14,18–21^. Using data from this two-year field study, results show that manipulating nitrogen fertilization, grain sorghum cultivars and in-furrow and foliar insecticide applications can help and synergistically suppress *M. sorghi* infestations and improve grain yield in sorghum production. Although aphid infestation was generally low in 2023 due to reasons discussed below, high and medium nitrogen fertilization, in-furrow and foliar insecticide applications and to a lesser extent, resistant grain sorghum cultivar, preserved yield compared to low nitrogen fertilization, untreated with foliar insecticide, and susceptible grain sorghum plots.

Similar to previous studies^5,18,19^ aphid populations across all three nitrogen fertilization treatments in both the susceptible (DKS53-53) and the resistant (DKS37-07) cultivars in 2022 peaked between three to four weeks from the date of first infestation. Although the low nitrogen fertilized susceptible sorghum cultivar had high aphid numbers, the high nitrogen fertilized plots had generally higher aphid density across treatment combinations as was also evident in the higher CID values in high nitrogen fertilized plots. Higher aphid density on grain sorghum treated with high nitrogen fertilization in the current study is consistent with the conclusions of previous reports that linked high nitrogen fertilization in plants with improved survival, developmental and population growth rate in insects including aphids^29,30,34,35,38^. Wilson et al.^35^ demonstrated increased density of *M. sorghi* in grain sorghum plots that received high and medium nitrogen fertilization. In a greenhouse experiment, Lama et al.^34^ showed a positive influence of nitrogen fertilization on developmental and growth rate of *M. sorghi* on susceptible grain sorghum (DKS38-38). While a plethora of studies including our previous reports demonstrate positive links between increased nitrogen and insect performance as well as population growth rate, studies reporting the negative effects of high foliar nitrogen on insect performance metrics are not uncommon^28,39,40^. For example, Zehnder and Hunter^28^ found that *Aphis nerii* (Hemiptera: Aphididae) per capita population growth rates on *Asclepias syriaca* (Apocynaceae) were highest at medium leaf nitrogen concentrations, while high nitrogen fertilization was linked with a decrease in growth rate. Similarly, Tao et al.^40^ showed that high foliar nitrogen concentrations in milkweed were correlated with a decrease in the population growth rate of the larvae of *Danaus plexippus*. The excess nitrogen in insect diet may be energetically costly as excreting and storing excess nutrients may impose some metabolic costs leading to slower growth, reduce reproduction and reduced population growth rates in insect herbivores^40,41^.

The early population collapse of *M. sorghi* on July 13th, 2022, and the double peak in aphid population was likely attributed to weather events. The *M. sorghi* population collapse in mid-July of 2022 on both sorghum cultivars and fertilization combinations coincided with two heavy rainfall events that amounted to 42 mm between 13 and 14th July 2022. Severe weather events, especially intense rainstorms and strong winds, have been found to decimate or eliminate cereal aphid populations including, *Sitobion avenae* (F.), *Metopolophium dirhodum* (Walk.), *S. fragariae* (Buck.) and *Rhopalosiphum padi* (L.) on *Triticum aestivum* (L.) (Poaceae)^42–44^. For example, Uyi et al.^19^ linked heavy rainfall events with aphid population collapse in grain sorghum and forage sorghum in Tifton, GA and Florence, SC. Rainfall events did not seem to explain the extremely low population of aphids in 2023 as the total rainfall during June to September of 2022 was more than that of the same period in 2023 (Table 3). Thus, it is possible that the persistent low winter temperatures of − 2 to − 9 °C for 6 days (between 23 and 28 December 2022) (Fig. 9) may have decimated the overwintering *M. sorghi* population such that the few individuals that survived migration to the sorghum plants were unable to reproduce rapidly and reach economic threshold where they can cause any economic damage in grain sorghum in the 2023 study. Persistently low temperature is known to negatively impact survival, development and reduced population growth rate in insects including aphids (e.g.^8,45^).

The higher aphid density on the susceptible cultivar (DKS53-53) relative to the resistant cultivar (DKS37-07) is consistent with previous studies reporting fewer aphids colonizing DKS37-07^13,14,34,35^. These results suggest that aphid resistant cultivars remain central to developing IPM program for the control of *M. sorghi* in the United States. Although the mechanism of resistance to *M. sorghi* was not well understood, resistant sorghum cultivars such as DKS37-07 are known to possess traits that contribute to antibiosis, antixenosis and tolerance^10^.

Relative to the control treatment, a single foliar application of flupyradifurone across all nitrogen fertilization levels and cultivars reduced aphid population to near zero in 2022. Other reports have demonstrated the efficacy of foliar application of flupyradifurone on both resistant and susceptible grain sorghum cultivars^13,21,35^. Plots that received foliar insecticide application did not show a resurgence except in susceptible cultivar that received low and high nitrogen fertilization. Relative to control treatment, in-furrow insecticide application clearly suppressed aphid numbers across nitrogen fertilization treatments and cultivars except in the medium and high fertilization treatments on susceptible cultivar. Uyi et al.^46^ and Uyi et al.^19^ documented the efficacy of in-furrow applications on susceptible grain sorghum and forage sorghum; aphid numbers were always less than 20 per leaf throughout the study in 2020 and 2021. The noticeably high aphid numbers on the susceptible cultivar that received medium and high fertilization treatments suggest that the applied in-furrow insecticide was losing efficacy by mid-August. Despite this, in-furrow applications of flupyradifurone are the most efficient and consistent method to manage *M. sorghi* infestations in grain sorghum especially within the context of IPM where the goal is to reduce foliar insecticide applications and manipulate other agronomic practices to reduce *M. sorghi* infestation and preserve grain yield^47^.

Irrespective of insecticide treatments, the higher CID values in low and high nitrogen fertilization treatments compared to the intermediate nitrogen fertilization treatment clearly showed that aphid infestations were more intense in low and high nitrogen fertilization plots. Wilson et al.^35^ found somewhat inconsistent influence of nitrogen fertilization on *M. sorghi* density in their study; increased aphid density was evident in the fertilized plots in Louisiana while nitrogen addition did not significantly influence aphid density in South Carolina. While that study was well replicated and executed, it must be noted that they did not compute CID values. Cumulative insect days (CID) differ from population density because CID simultaneously accounts for aphid abundance and duration of infestations to compute severity of infestations. Relative to untreated plots, the significantly lower CID values in insecticide treated sorghum across all nitrogen fertilized plots further confirms the efficacy of foliar and in-furrow insecticide applications as has been reported by previous studies (e.g.,^13,19^. The significantly lower CID values in resistant cultivar across all insecticide treated plots and nitrogen treated plots further demonstrate the importance of host plant resistance in managing *M. sorghi* infestation to reduce aphid population to levels where they do not cause economic damage to grain sorghum.

Expectedly, high and intermediate levels of nitrogen fertilization preserved yield in the 2022 trial; however, the influence of fertilization was more influential in the 2023 study, given that the condition of aphid infestation did not reach economic threshold, where medium and high nitrogen fertilization preserved yield by more than twofold in both resistant and susceptible cultivars. Although nitrogen fertilization has been linked with improved yield, studies reporting inconsistent or counterintuitive results in *M. sorghi* have been documented (see^35^). For example, Wilson et al.^35^ reported a twofold increase in grain sorghum yield resulting from fertilization in Louisiana, while in a similar study in South Carolina, nitrogen addition did not significantly increase grain sorghum yield. The lack of significant difference in grain sorghum yield between the medium and high nitrogen fertilization treatments in 2022 suggests that growers may benefit from the applying intermediate nitrogen fertilization in grain sorghum production thereby avoiding potentially excess nitrogen. The fact that the resistant cultivar only preserved yield in the low nitrogen fertilization plots in 2022, suggests that cultivar may not have had substantial influence on yield as has been reported in previous studies (e.g.,^13,14^). Relative to control treatment, insecticide application significantly improved yield across all nitrogen fertilization treatments except in the 2023 study where insecticide did not show a significant influence on grain yield. The positive link between insecticide application and grain sorghum yield is well documented^13,14,19^, however, previous studies have surprisingly also demonstrated inconsistent results between insecticide application and grain sorghum yield (e.g.^13,14^). The lack of a difference in grain yield between untreated and in-furrow insecticide treated plots in the 2023 study suggest that insecticide application may not have been the only limiting factor for yield in 2023. Also, the aphid population in 2023 may have been too low for the effect of insecticide application on yield to be detected. The findings strongly suggest that growers will not benefit from applying insecticides when *M. sorghi* infestations are low or below the economic threshold levels. Beyond aphid infestations, several factors such as plant disease, water stress and even bird damage prior to harvest can confound yield results in grain sorghum trials (e.g.,^19^). The less consistent impact of insecticide (in-furrow and foliar) application on grain sorghum yield implies that a combination intermediate nitrogen fertilization, resistant cultivar and in-furrow insecticide application in grain sorghum may be key to improving grain yield by growers in southeastern USA.

Because *M. sorghi* poses a serious challenge to grain sorghum production, and sometimes causes 100% yield loss to growers, comprehensive studies such as this are critical to refine IPM approach in grain sorghum production in the USA. Despite the high severity of aphid infestation in high fertilization plots, this research demonstrated that intermediate and high nitrogen fertilization singly and in combination with resistant sorghum cultivar and insecticide application significantly preserved grain yield in the 2022 study and to a lesser extent in the 2023 study. These data suggest that intermediate application of nitrogen in combination with planting resistant sorghum cultivar with good insecticide advice on and/or timing of insecticide application may be very critical in suppressing *M. sorghi* infestations and improving grain yield in sorghum production in southern USA. Therefore, studies manipulating and integrating a variety of grain sorghum production and pest management practices such as planting resistant grain sorghum cultivars, application of intermediate nitrogen fertilization, applying foliar or in-furrow insecticides or manipulating planting date to improve grain yield should be considered as an integral part of an IPM framework for managing *M. sorghi* in grain sorghum production. As an invasive pest, *M. sorghi* population requires integrated pest management practices, incorporating physical, cultural, and biological control methods^48^, with chemical control reserved as a last resort.

## Supplementary Information

Supplementary Information.

## Data Availability

All relevant data are within the paper.
