# Supplementary material for: Effects of insecticide use, host plant resistance, and nitrogen fertilization on the density of Melanaphis sorghi and the production of grain sorghum
Source: Sci Rep. 2025 Apr 9;15:12139. doi: 10.1038/s41598-025-96942-3 (PMC11982185; doi:10.1038/s41598-025-96942-3)
Supplement: Supplementary file 1 — Supplementary Information. [file 41598_2025_96942_MOESM1_ESM.pdf]

Supplementary Table 1. Soil test index before the study was conducted.

Phosphorus (P)	Potassium (K)	Calcium (Ca)	Magnesium (Mg)	Zinc (Zn)	Manganese (Mn)	Soil pH
18.60 kg/acre	47.14 kg/acre	36.43 kg/acre	32.22 kg/acre	0.78 kg/acre	1.93 kg/acre	6.63

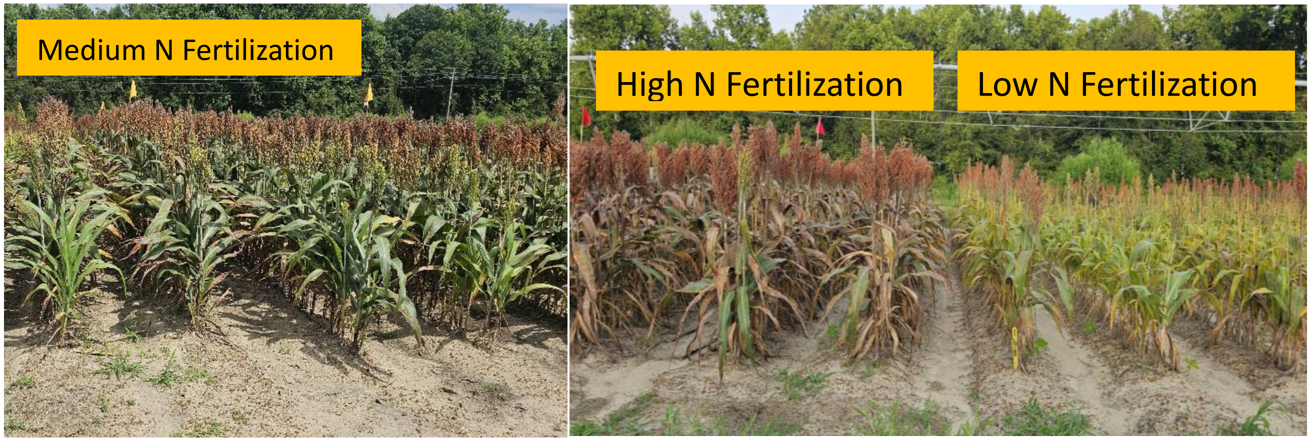

Supplementary Figure 1. Pictures of grain sorghum plots showing the different N. Fertilization treatments.
